# Stressful life events and resilience in individuals with and without a history of eating disorders: a latent class analysis

**DOI:** 10.1186/s40337-023-00907-8

**Published:** 2023-10-16

**Authors:** Selma Øverland Lie, Line Wisting, Kristin Stedal, Øyvind Rø, Oddgeir Friborg

**Affiliations:** 1 Regional Department for Eating Disorders, Division of Mental Health and Addiction, Oslo University Hospital, P.O. Box 4956, 0424 Nydalen, Oslo, Norway; 2 Institute of Clinical Medicine, University of Oslo, Oslo, Norway; 3 Faculty of Health Sciences Department of Psychology, UiT - The Arctic University of Norway, Tromsø, Norway

**Keywords:** Eating disorders, Bullying, Stressful life events, Resilience, Emotion regulation, Latent class analysis

## Abstract

**Background:**

Eating disorders (EDs) are associated with a range of stressful life events, but few have investigated protective factors that may affect these associations. The current study used mixture modelling to describe typologies in life stress exposure and availability of protective resources in individuals with and without eating disorders (EDs).

**Methods:**

A case – control sample (*n* = 916) completed measures of stressful life events, resilience protective factors, emotion regulation, and symptoms of EDs, depression and anxiety. We conducted latent class analyses to identify subgroups of stress exposure and profile analyses of emotional regulation and resilience. The resulting two latent variables were combined to explore effects on ED status and symptomatology, depression, and anxiety as distal outcome variables.

**Results:**

We identified four classes of stressful life events (generally low, some abuse/bullying, sexual/emotional assaults, and high adversity). For protective resources, we identified six profiles that ranged from low to higher levels of protection with variations in social/family resources. The latent protection variable contributed more strongly to the distal outcomes than the latent stress variable, but did not moderate the latent stress and distal outcome variable relationships. Profiles characterized by lower protective resources included higher proportions of individuals with a lifetime ED, and were associated with higher scores on all symptom measures.

**Conclusions:**

Intra- and interpersonal protective resources were strongly associated with lifetime EDs and current mental health symptom burden after accounting for stressful event exposure, suggesting protective factors may be useful to target in the clinical treatment of patients with ED.

## Abstract (plain language summary)

Previous studies have highlighted risk factors for eating disorders such as experiencing stressful or traumatic events. Protective resources, on the other hand, have received less attention. Factors such as resilience and emotion regulation are associated with eating disorders and could be important protective factors against severe illness in the presence of before mentioned risk factors. This study investigated levels of both potential risk (stressful life events) and protective (resilience and emotion regulation) factors in individuals with and without eating disorders. We found that individuals with low levels of protective resources showed more symptoms related to eating disorders, depression, and anxiety, suggesting that promoting protective factors could be an important avenue for future research, and a potential target for prevention and intervention efforts.

## Background

Among the factors found to increase risk of eating disorders EDs; [1, 2], are adverse events such as childhood trauma (e.g., abuse, neglect), sexual assault, war related trauma, and bullying [3–7]. Additionally, exposure to multiple or repeated trauma or adverse experiences is associated with a cumulative risk of negative health effects [8].

However, the individual differences in response to life stressors may be substantial. A concept of interest in this regard is resilience, representing a multi-dimensional construct denoting the ability to sustain relatively normal functioning despite exposures to significant adversity or trauma [9]. Resilient people often show increased flexibility and capacity to cope with life troubles, and have available protective resources that vulnerable individuals more often lack [10, 11]. In addition to resilience, emotion regulation is a multidimensional concept that plays an important role across the ED subtypes, and is associated both with disorder characteristics and prognosis [12–14]. Efficient emotion regulation is therefore another protective resource related to the other factors captured by the concept of resilience. Protective resources that promote adaption seem to cluster around three over-arching domains; a) psychological and dispositional attributes, b) family support and cohesion, and c) external support systems [11, 15].

Lower levels of resilience-related factors have been associated with increased risk of depression, anxiety, and other mental health indicators [16]. Good access to internal and external resilience factors may help mitigate risks, such as the effect of stressful life events (SLEs), by increasing the propensity to actively manage stressful incidents or new situations. Individuals with EDs often find it difficult to handle change and novelty, and are often socially isolated, maintain few friends, and in many cases family dynamics have been disrupted [17]. In addition, difficulties with inhibition, impulsivity, adapting to changes, and cognitive flexibility are common across the spectrum of EDs, and might therefore be related to the presence and the ability to utilize resilience factors when faced with psychological stressors or adversity in general [18–20].

Thus, examining specific resilience factors among individuals with EDs may aid in understanding contributions to risk and protection in this population, and it is of clinical interest to study factors that may influence the development or course of the illness within a transdiagnostic perspective. In line with this, exploring both ED specific symptoms and more general psychopathological features related to depression and anxiety furthers the transdiagnostic approach and explores differences in clinical presentations not confined solely to ED characteristics.

A few studies have attempted to characterize resilience among individuals with EDs. A recent study by Fergerson and Brausch [21] found that the effect of trauma on ED behaviors in women who had experienced sexual assault was significantly mediated by resilience as a measure of the ability to recover from adversity. In a 1-year longitudinal study of ED patients [22] examining resilience as self-acceptance and personal competence, improvements in quality of life and eating attitudes was seen among those with high resilience. Another recent study by Robert, Shankland [23] observed that resilience may be relevant for the prognosis of EDs, as higher resilience yielded a better chance of recovering from ED [23].

In contrast to the above studies that have used conventional regression analytic methods, few studies have adopted person-centered analytic approaches that explore how risk and protection factors may be related to EDs in disparate ways in different substrates of a heterogeneous sample. The use of latent class analysis LCA: e.g., [24], which is part of the broader family of latent mixture modeling approaches [25], have become increasingly popular for identifying such patterns. The LCA searches for attributes that subgroups of individuals share on selected indicator variables and make them alike. It estimates latent class parameters that describe the probability each individual has of belonging to a specific class (or group), and assign the individual to the class with the highest probability. This process identifies individuals that tend to cluster together, thus maximizing homogeneity within classes and heterogeneity between classes. By reporting the probabilities individuals in specific classes have of endorsing specific indicator values, the nature of the extracted classes may be described [26]. A LCA approach may be highly useful in studies on EDs for identifying and describing different patterns of symptom expressions that may be clinically meaningful within the transdiagnostic model. It also provides further opportunities for exploring if certain risk/protective patterns are more pronounced within certain substrates of individuals with EDs, and what the nature of these might be both within and between samples with ED pathology and healthy controls [27]. Moreover, it enables an analysis of how these patterns relate to disorder characteristics by adding “distal” outcome variables. In addition to ED symptoms and diagnosis, we also explore the relationship between risk/protective patterns and commonly co-morbid and more general features of depression and anxiety. Finally, this method allows for covariates both for conventional adjustment purposes, and, for predicting latent class memberships.

The objective of this study was to use mixture modeling to explore latent clustering in two sets of indicator variables: (1) history of exposure to stressful life events (SLEs), and (2) response patterns in protection/vulnerability data (resilience resources and emotion regulation) in a sample of individuals with and without a lifetime ED. We first examined the nature of the latent profiles related to the SLE and the protective data separately, and then how these two latent domains (SLE and protection/vulnerability) separately and in combination correlate with expressions of EDs, depression, and anxiety symptomatology. Finally, we tested if any latent protection/vulnerability classes moderated the relationship between the SLE classes and psychopathology scores.

## Methods

### Study setting and design

The current study was a part of the cross-sectional case control study Eating Disorders – Genes and Environment (EDGE), investigating risk and protective factors for EDs. Individuals (above 16 years) with and without a lifetime history of EDs were eligible, and the final study sample represents a convenience sample of cases and controls. All data were collected online between June 2019 and January 2020. The study and the procedures were approved by the Norwegian Regional Committee for Medical and Health Research Ethics (#2017/0606), and was conducted in accordance with ethical guidelines and regulations.

## Participants and procedures

A total of 916 individuals (95% female, age *M* 29.6, *SD* 10.7 years) participated. Individuals were classified as either cases (*n* = 495) or controls (*n* = 395) according to lifetime history of EDs. We were unable to determine ED status for the remaining 26 participants, and these were included in the whole sample analyses but not the direct case – control comparisons. All participants completed an online assessment including the study measures and descriptive information, and provided informed consent electronically using the Norwegian secure login system BankID. Each item had to be completed in order to limit missing data. The complete study materials took between 20 and 60 min to finalize, and participants could enter in to win an iPad if they wished to do so. All data was stored on a platform for sensitive information hosted by the University of Oslo.

Recruitment of both case and control participants was achieved through online social media platforms (Facebook and Twitter), and flyers and posters at Norwegian universities. Posts on websites for ED user organizations and flyers at psychiatric clinics across the country specifically targeted individuals with an ED history. The study was advertised as an investigation of stressful life events and eating disorders. Further details on the recruitment and study procedures have been described previously [28–30]. Due to coding error, one participant was excluded from all LCA analyses resulting in a sample of 915 individuals for all LCA/LPA models.

## Measures used to estimate latent classes/profiles (LCA/LPA)

*Stressful Life Events (SLEs).* Exposure to SLEs was recorded with the *Stressful Life Events Screening Questionnaire* SLESQ; [31] covering 12 events: disease (serious/life threatening), accident (serious/life threatening), assault (e.g., physical attack or robbery), bereavement (loss of a close relative, partner or friend), rape, other sexual assault (unwanted sexual contact/touching), childhood physical abuse (< 18 years of age), adult physical abuse (> 18 years of age), emotional abuse, threats with weapon or by force, witnessing violence (seeing another person being hurt, abused, or dying), or other events (representing a threat to life, health, or safety). We also included one item assessing exposure to bullying during school age (6–18 years). This was based on responses from the *Retrospective Bullying Questionnaire* RBQ; [32], and we coded bullying as present according to guidelines in the original measure and our previous publication [28, 32]. All measures of SLEs were thus based on retrospective recall of past events.

*Resilience Scale for Adults (RSA).* The RSA is a self-report measure of resilience resources covering two over-arching domains: intra-personal and inter-personal protective factors. The RSA uses 33 items that are scored on a seven-point semantic differential response format [33], and assesses six protective factors [15, 34]: perception of self, planned future, social competence, and structured style (intrapersonal domain), and family cohesion and social resources (interpersonal domain). Higher scores on the RSA predict less psychiatric symptoms following stressful exposures [35]. Subscale scores are calculated as the average of the subscale item scores. In the present study, subscale scores were transformed to a 0–100 range as the RSA was used in combination with the DERS-SF scale (also transformed to a 0–100 range) for conducting latent class analyses. The subscale “structured style” was not included in these analyses as it has consistently performed less well in terms of construct validity and item score reliability [36].

*The difficulties in emotion regulation scale – short form (DERS-SF).* The 18-item DERS-SF [37] was used to assess emotion regulation deficits. The items were scored on a Likert scale (1-almost never to 5-almost always) and summed to obtain a total score. The DERS has been translated and validated for use in Norwegian samples [38], and the Cronbach’s alpha was high in the current study (*α* = 0.93). The DERS represents a transdiagnostic vulnerability factor, as disordered emotional regulation is common across eating disorder diagnoses [39, 40]. Scores were transformed to a 0–100 range, as was done for the RSA, and scores were reversed so that high scores indicated better functioning to match the RSA. DERS in the current study was therefore used as a protection factor, as the absence of emotion regulation difficulties was interpreted as a positive resource.

## Measures used for outcomes and covariates

*ED100K.* The self-report measure ED100K was used to assess lifetime history of the three main EDs anorexia nervosa (AN), bulimia nervosa (BN), and binge-eating disorder (BED) and was used to classify cases and controls [41]. The measure identified lifetime EDs based on presence and severity of symptoms and behaviors according to the DSM-5 diagnostic criteria [42]. Individuals who did not fulfil criteria for an ED at any point in their lifetime were classified as controls. Only criteria for AN, BN, and BED were used and therefore the presence of other EDs were not assessed in the sample. The measure has been previously validated and shown to provide accurate identification of EDs compared with diagnostic interviews with good positive (0.85–1) and negative (0.77–1) predictive validity [41].

*Eating Disorder Examination-Questionnaire (EDE-Q).* The EDE-Q is a 28-item scale measuring the presence and severity of ED symptoms and behaviors in the past 28 days [43]. A validated Norwegian translation was used [44]. Items are scored on a 7-point scale (from “0 – no days” to “6 – every day”). Scores from the individual items are summed and averaged to obtain a global score. In a Norwegian setting, a global EDE-Q cut-off score of > 2.5 has been found to successfully discriminate between clinical and non-clinical populations [45]. The EDE-Q had a satisfactory Cronbach’s alpha in our sample (*α* = 0.97).

*Generalized anxiety disorder (GAD) scale 7*. The 7-item GAD scale [46] was used to assess anxiety symptoms in the last 14 days. Each item was scored on a Likert scale (0 = not at all to 3 = nearly every day), and item scores summed up to achieve a total score. Scores ≥ 10 are considered to be in the clinical range [46]. A validated Norwegian version was used [47], and we obtained a satisfactory Cronbach’s alpha (*α* = 0.91).

*Patient health questionnaire (PHQ-9).* The PHQ-9 [48] was used to assess severity of depressive symptoms in the last 14 days. The scale consists of nine items scored on a Likert scale (0 = “not at all” to 3 = “nearly every day”) and summed to achieve a total sore, with scores ≥ 10 considered to be in clinical range. The PHQ-9 has been deemed appropriate for research purposes in Norway [49], and the Norwegian translation has acceptable psychometric properties [30]. The Cronbach’s alpha in our sample was satisfactory (*α* = 0.91).

## Statistical analysis

Descriptive statistics, correlations between study measures, and comparisons of means using Welch *t*-tests were conducted in R version 4.1.3 [50]. We conducted all latent variable analyses in Mplus 8.7 [51].

*Latent class analyses:* To identify typical patterns of exposure to the stressful life events (SLEs), a LCA was conducted based on the 13 dichotomously scored SLE variables (12 SLESQ items and one RBQ item). Since these represented discrete risk factors (scored 0-no or 1-yes), a single latent threshold parameter was estimated for each SLE variable for expressing the probability in terms of log-odds of a case belonging to each of the latent classes given their indicator score. Based on these estimates, a posterior probability was estimated for assigning the case to the class with the highest probability [27]. The number of latent classes fit to the data were continuously increased until an optimal class structure could be decided.

*Latent profile analyses:* The protection/vulnerability indicators (RSA/DERS) were continuous variables (score range 0–100), thus requiring an additional variance parameter that may require constrictions (e.g., equal variance across classes) to avoid convergence problems. The five RSA subscale scores and the DERS total score were rescaled to a common 0–100 range, and the DERS was reversed so that a low versus high score on any scale indicated lower versus better functioning, respectively. For simplicity, we use the term “protective resources” consistently throughout the manuscript when referring to the analysis based on these variables. We extracted an increasing number of profiles until further improvements in model fit abated.

*Model fit:* Both mixture models were estimated based on the entire sample, thus maximizing heterogeneity and enabling extraction of classes/profiles that are more sensitive to clinical deviations from normality. The log-likelihood function was estimated with the maximum likelihood function using robust errors (MLR). To avoid converging on local maxima, the number of random starts was adjusted upwards to achieve replication of the lowest log likelihood value for the estimated parameters [24]. To decide on the most appropriate model, the Bayesian Information Criterion BIC; [52] and the sample-size adjusted BIC SABIC; [53] were examined with lower values indicating better fit. In addition, the Bootstrapped Likelihood Ratio test BLRT; [54] indicates whether a *k*-class model yields significantly better fit than the *k*-1 class (simpler) model according to the *p*-value. Entropy is reported as a measure of the precision of the latent classifications ranging from 0-low to 1-high. Simulation studies suggest that the BIC and BLRT perform best for deciding the number of latent classes or profiles to extract [55]. In addition, we considered interpretability and differentiation of the latent class/profile solutions.

**Fig. 1:**
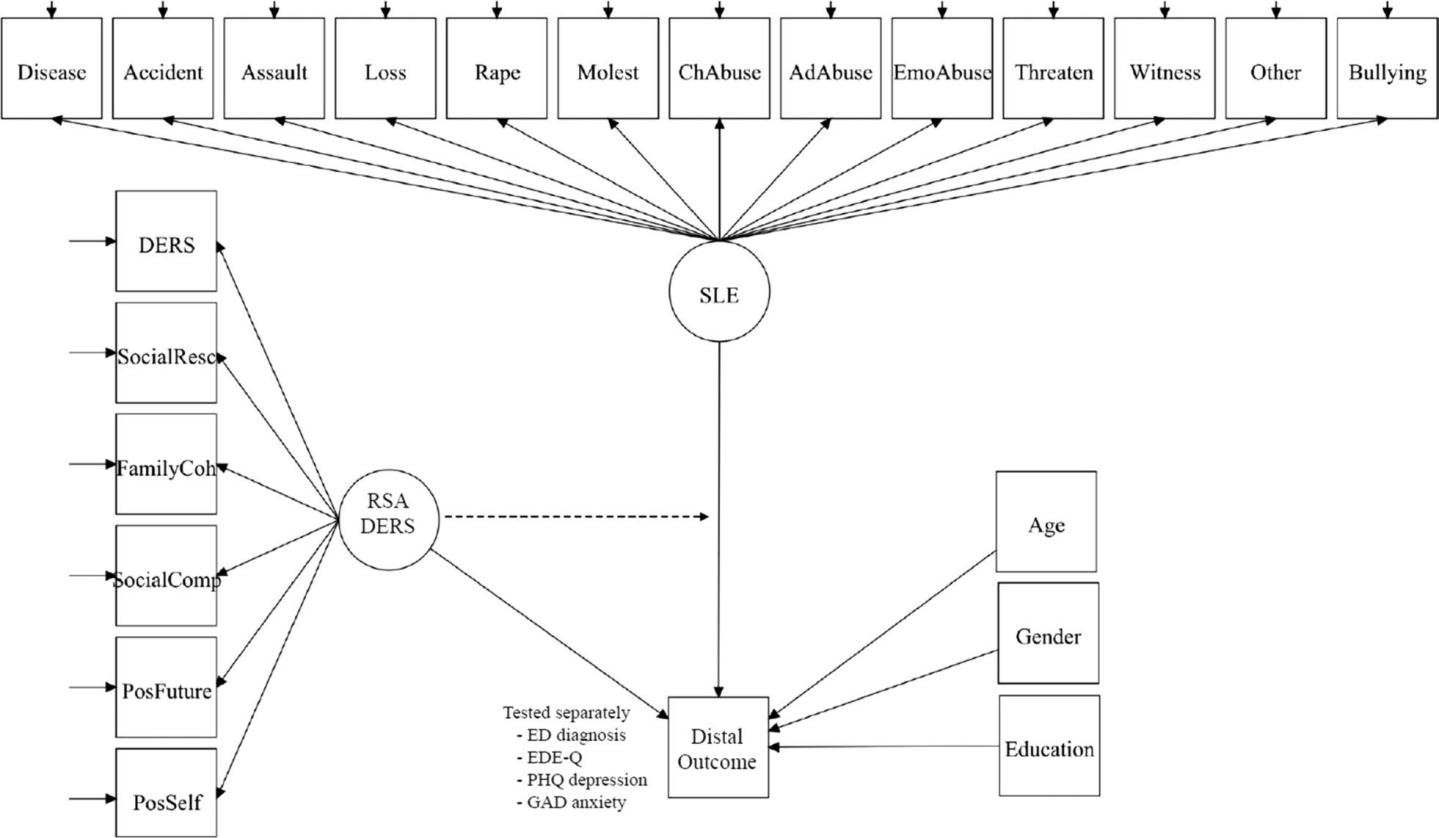
The Conceptual Latent Variable Model. *Notes:* DERS = Difficulties in Emotion Regulation Scale; EDE-Q = Eating Disorder Examination-Questionnaire; GAD = Generalized Anxiety Scale-7; PHQ = Patient Health Questionnaire-9; RSA = Resilience Scale for Adults

After deciding the number of latent classes and profiles, both were included as predictors (see Fig. 1) of the distal outcome variables assessing ED diagnosis (yes/no), EDE-Q symptom score, PHQ-depression score, and GAD-generalized anxiety score. Age, gender and education were added as covariates. The final joint model with both latent variables included (SLE and protective resources) together with the covariates for predicting the distal outcome scores, were based on the logit parameters for the within-class separations as devised by Asparouhov and Muthén [56]. This avoids substantial re-estimation of the within/between class parameters conditioned upon these extra variables. To examine the statistical significance of adding the two latent class/profile variables separately as main effects, combined and as an interaction effect, we applied the chi-square difference testing method with robust errors according to Satorra and Bentler [57].

The expected mean score of the four distal outcome variables were examined separately; hence, we reduced the alpha level to < 0.01. We added both latent variables together with a distal outcome variable, first without covariates (crude model) and subsequently with covariates included (adjusted model). The joint mixture model represents a two-way full interaction model since the expected mean outcome values are estimated as free in all 24 latent group combinations (4 SLE × 6 protection classes). To examine the significance of SLE and RSA-DERS as separate fixed effects, we used model constraints. The baseline model had the outcome mean values constrained as equal across all 24 group combinations, thus representing a simple intercept model. To test the fixed effect of the RSA-DERS factor, we allowed the outcome scores to vary between the six RSA-DERS profiles while constraining them equal across the SLE classes, and vice versa when testing the fixed effect of the latent SLE variable. If the log likelihood of this model improved significantly as compared to the log likelihood of the intercept model according to the chi-square difference test [57], it was considered significant. The significance testing of the SLE latent class variable was done comparably. We then examined the combined fixed effect of both latent variables. Since the RSA-DERS variable improved model fit the most, this model functioned as a comparison model to the addition of both latent variables. The model constrictions were similar as above for the RSA-DERS fixed factor but allowed for an additive SLE effect. The interaction model was compared to the model specifying both latent variables as combined fixed effects, but had no constrictions, thus freely estimating all 24 combinations. The final adjusted model included the covariates.

*Contingency analysis:* Based on the two latent variables, the nominal latent variable membership values were saved and analysed in SPSS 28 [58] with regard to their categorical associations using the cross-tabulation function. In case of an overall significant chi-square test, differences between column frequencies were followed up using standardized adjusted residuals, i.e., N(0,1). This provides a *z*-test based value that needs to surpass the square root of the critical chi-square value for the degrees of freedom of the test in question, i.e., *d.f.* = 15 [59]. Due to the large number of comparisons (60 cells), these z-tests were Bonferroni adjusted [59].

## Results

Descriptive statistics for the sample are reported in Table 1. In the whole sample, 35% met criteria for a current ED while 19% had a past history of an ED. The remaining 46% were treated as no-ED control cases. In the case group, 36% had a history of AN, 37% of BN/BED, and 27% of both AN and BN/BED. As expected, the ED group scored higher than the control group on all measures of psychopathology and emotion regulation difficulties (Table 1). The overall prevalence of stressful life events (81% vs 65%) and bullying (32% vs 19%) was higher in the ED compared to the control group, as previously reported [28, 29].

**Table 1:** Description of the Overall Sample (*N* = 916), and Separate by ED Case–Control Status

	Range	All *N* = 916	ED cases^a^ *n* = 495 (62% current ED)	No ED *n* = 395
		*M* (SD)	*M* (SD)	*M* (SD)
Age (yrs)	16–78	29.45 (10.62)	29.08 (9.76)	30.16 (11.66)
Current BMI	12.4–58.6	23.99 (6.18)	23.85 (7.29)	23.94 (4.41)
ED onset age (yrs)		−	15.09 (4.58)	–
EDE-Q	0–6	2.42 (1.74)	3.32 (1.54)	1.28 (1.26)
DERS-SF	18–90	48.76 (15.71)	55.37 (14.21)	40.27 (13.3)
PHQ-9	0–27	10.99 (7.20)	14.21 (6.71)	6.94 (5.67)
GAD-7	0–21	9.03 (5.71)	11.28 (5.45)	6.22 (4.69)
		*n* (%)	*n* (%)	*n* (%)
EDE-Q cut-off (> 2.5)^b^		427 (47%)	351 (71%)	62 (16%)
PHQ-9 cut-off (> 10)^b^		481 (53%)	363 (73%)	105 (27%)
GAD-7 cut-off (> 10)^b^		385 (42%)	290 (59%)	86 (22%)
Education				
primary school		111 (12%)	68 (14%)	40 (10%)
upper secondary		294 (32%)	170 (34%)	112 (28%)
university < 4 yrs		281 (31%)	152 (31%)	123 (31%)
university > 4 yrs		210 (23%)	91 (18%)	114 (29%)
other		20 (2%)	14 (3%)	6 (2%)
Gender				
female		875 (95%)	485 (98%)	365 (92%)
male		41 (5%)	10 (2%)	30 (8%)

^a^ED status could not be ascertained for *n* = 26 participants, therefore not all numbers in the “ED cases” and “No ED” columns sum to the number listed for the full sample (“All” column).^b^Cut-off scores indicate scores above substantial clinical symptoms that may require treatment

BMI = Body mass index (kg/m2); DERS-SF = Difficulties in Emotion Regulation Scale-Short Form; ED = Eating disorders, EDE-Q = Eating Disorder Examination-Questionnaire; GAD-7 = Generalized Anxiety Scale-7; PHQ-9 = Patient Health Questionnaire-9

## Differences in RSA scores for ED cases and controls

Cronbach’s alphas were satisfactory for most RSA subscales, except for “structured style” (Table 2), which had unsatisfactory values in both groups (cases and controls). The RSA global scores were significantly different between cases and controls, with a large effect size (*g* = − 0.87). For the subscales “perception of self”, “perception of future”, “social competence”, “family cohesion”, and “social resources”, the ED group scored significantly lower than the control group with medium to large effect sizes (*g*’s from − 0.5 to − 1.04). Table 2 shows the means, differences between groups, and effect sizes for all RSA scores.

**Table 2:** Mean Score Differences on the Resilience Scale for Adults with Respect to ED Case–Control Status

RSA subscale	Full sample *n* = 916 *M* (SD)	ED *n* = 495 *M* (SD)	Controls *n* = 395 *M* (SD)	*M* diff _95% CI_ Hedges’* g*_95%CI_	Cronbach’s *α* *t* (df)
Perception of self	3.81 (1.46)	3.21 (1.32)	4.56 (1.27)	− 1.34 _− 1.52 | − 1.17_ − 1.04 _− 1.18 | − 0.89_	0.87 15.42 _df=858_^***^
Perception of future	4.25 (1.72)	3.64 (1.63)	5.02 (1.51)	− 1.39 _− 1.59 | − 1.18_ − 0.88 _− 1.02 | − 0.74_	0.89 13.13 _df=869_^***^
Social competence	4.28 (1.32)	4.00 (1.34)	4.67 (1.21)	− 0.67 _− 0.84 | − 0.50_ − 0.52 _− 0.66 | − 0.39_	0.82 7.81 _df=875_^***^
Family cohesion	4.69 (1.45)	4.38 (1.49)	5.09 (1.30)	− 0.71 _− 0.89 | − 0.53_ − 0.50 _− 0.64 | − 0.37_	0.89 7.60 _df=881_^***^
Social resources	5.43 (1.15)	5.17 (1.20)	5.78 (0.99)	− 0.61 _− 0.75 | − 0.46_ − 0.55 _− 0.68 | − 0.41_	0.85 8.29 _df=887_^***^
Structured style	4.8 (1.22)	4.74 (1.22)	4.87 (1.21)	− 0.13 _− 0.29 | 0.03_ − 0.10 _− 0.24 | 0.03_	0.58 1.54 _df=849_
Global score	4.57 (1.00)	4.22 (0.97)	5.03 (0.86)	− 0.81 _− 0.93 | − 0.69_ − 0.87 _− 1.01 | − 0.74_	0.93 13.13 _df=877_^***^

^***^p < 0.001, *M* = mean, SD = standard deviation, *M* diff = mean difference between cases and controls, 95% CI = 95% confidence intervals, *t* = Welch t-test, df = degrees of freedom

ED = Eating disorder; RSA = Resilience scale for adults

## Correlations between RSA and other measures (EDE-Q, PHQ, GAD, and DERS)

All RSA scores except “structured style” were significantly negatively correlated with the ED symptom measure EDE-Q (range − 0.32 to − 0.60, *p*’s < 0.05). “Perception of self” and “planned future” showed the strongest correlations with EDE-Q total score. The RSA global score also correlated strongly negatively with the other symptom measures of PHQ depression (*r* = − 0.73, *p* < 0.001) and GAD anxiety (*r* = − 0.64, *p* < 0.001), as well as with DERS-SF emotion regulation difficulties (*r* = − 0.73, *p* < 0.001). Higher resilience protective scores thus implied a lower degree of current pathological symptoms. The RSA “structured style” subscale was weakly related to the symptom measures, which is in line with previous reports.

## Latent class analyses of stressful life events

The fit indices were inconsistent regarding the preferred number of latent classes, with the BIC favouring a 3-class solution, the SABIC 4 classes, and the BLRT 5 classes (Table 3). We preferred the 4-class solution as it balanced parsimony with sufficient class differentiation and interpretability. The 4-class solution provided the best SABIC, as well as a BIC close to the 3-class solution. The entropy was also better for the 4-class solution.

**Table 3:** Model Fit Indices for the Latent Class Analysis of Stressful Life Event Exposures

Classes	#param	LL	BIC	SABIC	Entropy	BLRT
1	13	− 5111.15	10,310.97	10,269.68	–	–
2	27	− 4597.86	9379.87	9294.12	0.789	1026.58^***^
3	41	− 4521.25	*9322.11*	9191.90	0.717	153.24^***^
**4**	**55**	**− 4488.28**	**9351.65**	***9176.98***	**0.777**	65.94^***^
5	69	− 4467.08	9404.75	9185.61	0.774	42.39^***^
6	83	*− 4452.35*	9470.76	9207.17	*0.810*	29.47

Best-fitting indices are given in *italics*, and preferred class solution in bold. # param = Number of parameters, LL = Log likelihood, BIC = Bayesian Information Criterion, SABIC = Sample size adjusted BIC, BLRT = Bootstrapped Likelihood Ratio Test. ^*^p < 0.05 ^**^p < 0.01 ^***^p < 0.001

Regarding class characterization, class #1 was the most prevalent (51%) and represented individuals reporting a low level of exposure to any kind of SLEs. Class #2 was less prevalent (27%) and was characterized by a heightened endorsement (compared to class #1) of SLEs related to childhood physical abuse, emotional abuse, molestation, and bullying, as well as a particularly high endorsement of unspecified events (76%). Class #3 (14%) mimicked class #2 but represented individuals who all (100%) had been exposed to sexual assaults. Class #4 (9%) describes a low prevalence but high adversity class representing individuals with a high probability of being exposed to a broad spectrum of adverse events, thus representing individuals with a considerable accumulation of stress burden. Table 4 shows the probabilities of endorsing the SLE items within each of the classes.

**Table 4:** Model Estimated Class-Specific Proportions of Stressful Life Event Exposure

	Class 1 *n* = 466 (51%)	Class 2 *n* = 247 (27%)	Class 3 *n* = 124 (14%)	Class 4 *n* = 79 (9%)
	Low exposure	Higher unspecified, physical/emotional abuse and bullying	Sexual assault class with high molestation	High and broad adverse exposure
Stressful life event	*pr*_95% *CI*_	*pr*_95% *CI*_	*pr*_95% *CI*_	*pr*_95% *CI*_
Disease	.058 _.032 | .084_	.163 _.106 | .220_	.084 _.019 | .149_	.219 _.106 | .332_
Accident	.026 _.006 | .046_	.112 _.065 | .159_	.029 _− .013 | .071_	.233 _.116 | .350_
Assault	.009 _.000 | .021_	.086 _.041 | .131_	.120 _.053 | .187_	.522 _.365 | .679_
Loss	.085 _.054 | .116_	.154 _.097 | .211_	.309 _.214 | .404_	.498 _.371 | .625_
Rape	.026_.000 | .055_	0 _.000 | .000_	1.000 _1.000 | 1.000_	.848 _.739 | .957_
Molested	.085 _.050 | .120_	.291 _.210 | .372_	.636 _.527 | .745_	.799 _.692 | .906_
Child physical abuse	.025 _.001 | .049_	.312 _.219 | .405_	.199 _.090 | .308_	.776 _.647 | .905_
Adult physical abuse	.014 _.000 | .032_	.120 _.069 | .171_	.153 _.052 | .254_	.615 _.474 | .756_
Emotional abuse	.077 _.030 | .124_	.484 _.395 | .573_	.491 _.345 | .637_	1.000 _1.000 | 1.000_
Threatened	.008 _.000 | .018_	.063 _.028 | .098_	.030 _− .004 | .064_	.450 _.291 | .609_
Witnessed	.004 _.000 | .018_	.135 _.082 | .188_	.096 _.031 | .161_	.550 _.389 | .711_
Other	.247 _.180 | .314_	.757 _.664 | .850_	.637 _.526 | .748_	.793 _.666 | .920_
Bullied	.078 _.039 | .117_	.367 _.288 | .446_	.471 _.348 | .594_	.632 _.509 | .755_

*pr* = Proportion of the sample, 95% *CI* = 95% confidence interval

## Latent profile analyses of protective resources

The LPA models of the protection scores modelled the indicator variances as free across profiles rather than fixed (equal) as they fit consistently better. No further than seven profiles were modelled due to a local maxima that could not be resolved (lack of replication of the log-likelihood estimate). Since the BIC improvement decelerated substantially after the 6^th^ profile, this was the candidate solution. A deceleration in fit improvement was also evident following extraction of 4 profiles; however, as the 6-profile solution contained some qualitative differences with regard to higher family and social resources in combination with lower personal resources that the 4-profile solution missed, the 6-profile solution was preferred due to best fit and a conceptual relevant differentiation (see Table 5 for fit indices).

**Table 5:** Model Fit indices for the Latent Profile Analysis of the RSA and DERS Indicator Variables

Profiles	#param	corr	LL	BIC	SABIC	Entropy	BLRT
Fixed variances							
1	12	0.829	− 25,070.65	50,223.13	50,185.02	–	–
2	19	1.098	− 24,104.85	48,339.28	48,278.94	*0.859*	1931.59^***^
3	26	1.292	− 23,839.10	47,855.53	47,772.96	0.840	531.49^***^
4	33	1.232	− 23,752.00	47,729.06	47,624.26	0.820	174.21^***^
5	40	1.181	− 23,624.59	47,521.98	47,394.95	0.814	254.82^***^
6	47	1.234	− 23,572.83	47,466.19	47,316.92	0.812	103.53^***^
7	54	1.249	*− 23,541.29*	*47,450.87*	*47,279.37*	0.804	63.06^***^
Free variances							
1	Same as fixed						
2	25	1.239	− 24,016.50	48,203.50	48,124.10	*0.868*	2108.29^***^
3	38	1.552	− 23,710.81	47,680.78	47,560.09	0.848	611.39^***^
4	51	1.161	− 23,572.93	47,493.69	47,331.72	0.847	275.75^***^
5	64	1.189	− 23,462.92	47,362.32	47,159.07	0.823	220.03^***^
**6**	**77**	**1.181**	**− 23,383.49**	**47,292.13**	**47,047.58**	**0.828**	158.86^***^
7 ^nr^	90	1.132	*− 23,334.84*	*47,283.49*	*46,997.66*	0.814	97.30 ^nr^

Best-fitting indices are given in *italics*, and preferred class solution in bold. #param = Number of parameters, corr = MLR scaling correction, LL = Log likelihood, BIC = Bayesian Information Criterion, SABIC = Sample size adjusted BIC, BLRT = Bootstrapped Likelihood Ratio Test, ^nr^ = not replicable. ^*^p < 0.05 ^**^p < 0.01 ^***^p < 0.001. DERS = Difficulties in emotion regulation scale, RSA = Resilience scale for adults

The descriptive nature of the protection profiles are presented in Table 6. Profiles #1 and #2 represented 30% of the sample and included individuals with good access to protective resources, of which the first profile had the highest probability for good adaptation capacity. Profile #3 and #4 constituted 42% of the sample representing individuals with a medium level of resilience resources, in which profile #3 was distinguished from profile #4 by better access to family and social protective resources. Profile #5 and #6 were less prevalent (27%) and were characterized by low availability of intrapersonal protective resources, of which profile #5 had better family and social resources than profile #6.

**Table 6:** Latent Profile Estimated Mean Scores for the Resilience Protection and DERS Emotional Regulation Scores

	Profile 1 *n* = 102 (11.1%)	Profile 2 *n* = 176 (19.2%)	Profile 3 *n* = 168 (18.3%)	Profile 4 *n* = 223 (24.3%)	Profile 5 *n* = 137 (15%)	Profile 6 *n* = 110 (12%)
Profile description	Well protected, high adaptability	Good protection / adaptability	Medium protection, high family / social resources	Medium protection	Low protection, moderate family/social resources	Low protection / adaptability
Subscales	*M*_95% CI_	*M*_95% CI_	*M*_95% CI_	*M*_95% CI_	*M*_95% CI_	*M*_95% CI_
RSA self	80.4 _77.3 | 83.4_	69.8 _66.7 | 72.9_	47.4 _43.1 | 51.7_	44.9 _39.7 | 50.2_	19.6 _15.4 | 23.9_	16.1 _12.9 | 19.3_
RSA future	89.9 _87.4 | 92.5_	79.3 _75.6 | 83.0_	58.9 _54.1 | 63.8_	48.8 _44.8 | 52.8_	31.3 _24.8 | 37.9_	14.0 _10.0 | 18.1_
RSA soc comp	79.6 _76.3 | 83.0_	65.1 _61.9 | 68.3_	59.6 _56.1 | 63.0_	50.5 _46.8 | 54.3_	43.1 _30.3 | 55.8_	31.8 _23.4 | 40.2_
RSA fam coh	89.2 _86.6 | 91.7_	66.2 _61.4 | 71.1_	81.0 _78.0 | 84.1_	44.8 _40.3 | 49.3_	62.8 _51.0 | 74.7_	33.7 _28.9 | 38.5_
RSA soc resc	96.2 _94.4 | 97.9_	81.7 _78.8 | 84.7_	87.8 _85.0 | 90.5_	62.8 _59.4 | 66.3_	74.0 _70.9 | 77.2_	43.4 _37.5 | 49.4_
DERS-SF^a^	84.8 _82.4 | 87.3_	77.6 _74.6 | 80.6_	59.8 _56.1 | 63.4_	53.2 _48.8 | 57.5_	38.3 _34.8 | 41.7_	28.1 _24.3 | 31.9_

95% confidence intervals are given in subscript. All scores were rescaled to a common 0–100 range with higher scores representing better protection/functioning. RSA self = positive perception of self, RSA future = positive planned future, RSA soc comp = social competence, RSA fam coh = family cohesion, RSA soc resc = social resources, DERS-SF = emotion regulation capability. ^a^DERS-SF score reversed from original so that higher scores indicate better emotion regulation abilities/less difficulties

## Associations between the latent SLE and protection variables

The nominal latent class and nominal latent profile categorizations were saved and subjected to a two-way contingency analysis, which was significant (χ^2^_df=15_ = 113.2, *p* < 0.001; moderate effect size Cramer’s V = 0.20). See Table 7 for the observed and expected cell observations (‘contingency’). The adjusted standardized z-test residual values indicate if placements in specific SLE classes are significantly more or less frequent than expected. The associative pattern showed that individuals in the less well protected RSA-DERS profile groups #4–6 are significantly more often (positive z-values) exposed to adverse events than individuals in the better protected groups #1–3 (negative z-values). The z-values were also significantly different for individuals in RSA/DERS profile #3 and profile #4, which shows that individuals from a family characterized by less cohesion are more often exposed to adverse events of SLE class #2 (physical/emotional abuse, and unspecified events) and class #4 (broader and generally higher level of adversity) than individuals from a family of high cohesion despite both groups having relatively comparable intrapersonal resources.

**Table 7:** Categorical Associations (Contingency Tests) Between SLE and RSA-DERS Latent Variables

	RSA-DERS latent profile groups						
SLE latent class		1	2	3	4	5	6
1	Contingency						
	Obs *n* / Exp *n*	79 / 51.3	109 / 89.4	106 / 85.4	84 / 113.3	61 / 69.6	26 / 55.9
	Adj resid	5.8_a_	3.3_a_	3.5_a_	− 4.5_b. c_	− 1.6_c_	− 6.1_b_
2	Contingency						
	Obs *n* / Exp *n*	13 / 27.3	37 / 47.5	34 / 45.4	77 / 60.2	42 / 37.0	44 / 29.7
	Adj resid	− 3.4_a_	− 2.0_a. b_	− 2.2_a. b_	2.9_c_	1.0_b. c_	3.3_c_
3	Contingency						
	Obs *n* / Exp *n*	5 / 13.7	19 / 23.9	21 / 22.8	30 / 30.2	27 / 18.6	22 / 14.9
	Adj resid	− 2.7_a_	− 1.2_a. b_	− 0.4_ab_	0.0_a. b_	2.3_b_	2.1_b_
4	Contingency						
	Obs *n* / Exp *n*	4 / 8.7	11 / 15.2	7 / 14.5	32 / 19.3	7 / 11.8	18 / 9.5
	Adj resid	− 1.8_ab_	− 1.3_abc_	− 2.3_b_	3.5_a. c_	− 1.6_a.b.c_	3.1_c_

*Contingency tests:* The null hypothesis of no categorical association between the SLE and RSA-DERS cells was discarded (χ^2^_df=15_ = 113.2, *p* < .001; Cramer’s V correlation = 0.20). Obs *n* / Exp *n* = observed / expected cell frequency, adjR = Adjusted standardized residual values. Subscript letters (e.g., _**a**_ and _**b**_) that are different on the same row between any two RSA-DERS profile columns indicate that these two proportions are significantly different (Bonferroni adjusted). DERS-SF = Difficulties in Emotion Regulation Scale-Short Form; RSA = Resilience Scale for Adults; SLE = Stressful Life Event

## Significance testing of the latent variables and the predictors

The RSA-DERS latent variable significantly explained the mean scores of all distal outcome, whereas the SLE latent variable contributed significantly in three of the four distal outcome models (see Table 8). The exception was GAD-7 anxiety, in which the latent SLE variable turned non-significant after adding the RSA-DERS latent variable. Since the interaction model did not reach significance in any of the distal outcome models, implying that the latent RSA-DERS factor did not modify the relationship between the latent SLE factor and any of the distal outcome variables, the interaction effect was omitted in the final distal outcome results as presented in Table 9.

**Table 8:** Significance Tests of the Full Model Parameters Together with Covariates and Distal Outcomes

Latent variables and covariates	ED diagnosis %		EDE-Q range 0–6		PHQ-9 depression range 0–27		GAD-7 anxiety range 0–21	
Chi-sq diff tests	Crude	Full model	Crude	Full model	Crude	Full model	Crude	Full model
SLE ^icept^	53.41 _df=3***_	50.56 _df=3_^***^	84.11 _df=3_^***^	80.97 _df=3_^***^	92.40 _df=3_^***^	34.21 _df=3_^***^	52.49 _df=3_^***^	13.39 _df=3_^**^
RSA-DERS ^icept^	211.99 _df=5***_	188.98 _df=5_^***^	360.92 _df=5_^***^	318.88 _df=5_^***^	663.01 _df=5_^***^	316.75 _df=5_^***^	445.55 _df=5_^***^	212.62 _df=5_^***^
Both latent vars ^SLE^	15.22 _df=3**_	12.52 _df=3_^**^	36.72 _df=3_^***^	31.97 _df=3_^***^	36.13 _df=3_^***^	18.55 _df=3_^***^	6.70 _df=3_	4.21 _df=3_
Interaction ^BOTH^	10.09 _df=15_	9.07 _df=15_	12.60 _df=15_	13.10 _df=15_	21.04 _df=15_	17.08 _df=15_	9.40 _df=15_	20.52 _df=15_
Covariates		*odds-ratio*		*beta*		*beta*		*beta*
Gender (0 = ♀,1 = ♂)		0.39^*^		− 0.715^***^	− 1.88^***^^a^	− 0.89	− 1.10 ^a^	− 0.42
Age (16–78 yrs)		1.01		0.006	− 0.02 ^a^	− 0.03^*^	− 0.02^a^	− 0.03^*^
Education (0–4)		0.86		− 0.121^*^	− 0.45^*a^	− 0.24	− 0.27^a^	− 0.14
EDE-Q (0–6)						1.36^***^		0.88^***^

^icept^ Addition of one latent variable compared with no variables (intercept only), ^SLE^ = Addition of the SLE latent variable compared to a model with RSA-DERS latent variable included, ^BOTH^ = Addition of the interaction effect (SLE*RSA-DERS) compared to a model with both latent variables included. Crude = no covariates, full model = adjusted for gender, age, and education. ^a^ Latent SLE/DERS factors were adjusted for gender, age and education in the crude column for PHQ-9 and GAD-7 as distal outcomes, and additionally adjusted for EDE-Q in the full model. ^*^ p < 0.05 ^**^ p < 0.01 ^***^ p < 0.001

**Table 9:** Distal Outcomes Associated with the Latent Class/Profile Memberships of SLE and RSA-DERS Latent Variables

	RSA-DERS latent profile groups						
SLE latent class		1 M_CI 99.9%_	2 M_CI 99.9%_	3 M_CI 99.9%_	4 M_CI 99.9%_	5 M_CI 99.9%_	6 M_CI 99.9%_
1	Distal outcomes						
	ED diagnosis %	18.8% _8.4% | 36.9%_	19.2% _10.0% | 33.5%_	46.7% _29.8% | 64.3%_	50.8% _34.3% | 67.1%_	83% _59.8% | 94.1%_	82.9% _57.2% | 94.6%_
	EDE-Q	0.73 _0.39 | 1.07_	0.94 _0.65 | 1.23_	2.12 _1.59 | 2.66_	2.08 _1.57 | 2.60_	3.67 _2.98 | 4.36_	3.69 _3.03 | 4.35_
	Depression PHQ-9	5.15 _3.93 | 6.36_	5.86 _4.82 | 6.90_	9.05 _7.72 | 10.38_	10.70 _9.07 | 12.32_	15.23 _12.68 | 17.79_	17.46 _15.58 | 19.33_
	Anxiety GAD-7	4.55 _3.50 | 5.60_	5.45 _4.48 | 6.42_	8.22 _6.56 | 9.89_	8.89 _7.09 | 10.68_	12.85 _10.39 | 15.31_	14.21 _12.29 | 16.14_
2	Distal outcomes						
	ED diagnosis %	30.2% _12.3% | 57.2%_	30.7% _14.4% | 53.8%_	62.0% _39.1% | 80.6%_	65.9% _46.9% | 80.8%_	90.1% _71.4% | 97.1%_	90.0% _70.7% | 97.1%_
	EDE-Q	1.11 _0.60 | 1.62_	1.32 _0.87 | 1.77_	2.50 _1.84 | 3.17_	2.47 _1.92 | 3.01_	4.05 _3.38 | 4.72_	4.07 _3.46 | 4.68_
	Depression PHQ-9	6.03 _4.40 | 7.65_	6.74 _5.31 | 8.17_	9.93 _8.32 | 11.54_	11.58 _9.73 | 13.43_	16.11 _13.60 | 18.63_	18.34 _16.49 | 20.18_
	Anxiety GAD-7	4.93 _3.38 | 6.47_	5.82 _4.41 | 7.23_	8.60 _6.65 | 10.54_	9.26 _7.37 | 11.15_	13.22 _10.66 | 15.79_	14.59 _12.61 | 16.57_
3	Distal outcomes						
	ED diagnosis %	35.8% _13.3% | 67.0%_	36.4% _15.9% | 63.5%_	67.9% _41.8% | 86.1%_	71.4% _46.9% | 87.6%_	92.2% _73.7% | 98.0%_	92.1% _71.3% | 98.2%_
	EDE-Q	1.45 _0.85 | 2.05_	1.66 _1.11 | 2.22_	2.85 _2.15 | 3.54_	2.81 _2.14 | 3.48_	4.39 _3.71 | 5.07_	4.41 _3.72 | 5.10_
	Depression PHQ-9	6.42 _4.41 | 8.43_	7.13 _5.31 | 8.95_	10.32 _8.33 | 12.31_	11.97 _9.60 | 14.35_	16.51 _13.83 | 19.18_	18.73 _16.55 | 20.91_
	Anxiety GAD-7	4.20 _2.47 | 5.92_	5.09 _3.42 | 6.76_	7.87 _5.82 | 9.91_	8.53 _6.18 | 10.88_	12.49 _9.96 | 15.03_	13.86 _11.74 | 15.97_
4	Distal outcomes						
	ED diagnosis %	32.9% _10.3% | 67.7%_	33.5% _12.1% | 64.7%_	65.0% _33.5% | 87.2%_	68.7% _40.8% | 87.5%_	91.2% _66.8% | 98.2%_	91.1% _67.5% | 98.1%_
	EDE-Q	1.59 _0.87 | 2.31_	1.80 _1.13 | 2.48_	2.99 _2.18 | 3.79_	2.95 _2.27 | 3.63_	4.53 _3.62 | 5.45_	4.55 _3.77 | 5.34_
	Depression PHQ-9	7.63 _5.20 | 10.05_	8.34 _5.95 | 10.73_	11.53 _9.00 | 14.06_	13.18 _10.70 | 15.65_	17.71 _14.43 | 21.00_	19.94 _17.35 | 22.52_
	Anxiety GAD-7	5.61 _3.52 | 7.70_	6.51 _4.54 | 8.48_	9.28 _6.83 | 11.74_	9.95 _7.71 | 12.18_	13.91 _10.77 | 17.05_	15.27 _12.83 | 17.72_

*Distal outcomes*: *M*_CI 99.9%_ = Estimated mean score and 99.9% confidence intervals based on the final fully adjusted latent mixture model (see Table 8). ED distal outcome variables were covariate adjusted for age, gender and education, whereas PHQ depression and GAD anxiety were in addition adjusted for the EDE-Q case–control variable. ED diagnosis % = Proportion of individuals assigned a diagnosis of eating disorder. Post-hoc tests of the RSA profile column differences within the same SLE latent class with alpha lowered to < .001 due to the large number of tests. The post-hoc comparisons yielded similar conclusions for all distal outcome variables with the following column differences as significantly different: 1 < 3–6, 2 < 3–6, 3 < 5–6, 4 < 5–6 (e.g., 1 < 3–6, reading that column 1 estimated mean was significantly lower than the estimated means of column 3, 4, 5 and 6)

ED = Eating Disorder, EDE-Q = Eating Disorder Examination-Questionnaire (cut-off value > 2.5); DERS-SF = Difficulties in Emotion Regulation Scale-Short Form; GAD-7 = Generalized Anxiety Scale-7 (cut-off value > 10); PHQ-9 = Patient Health Questionnaire-9 (cut-off value > 10); RSA = Resilience Scale for Adults; SLE = Stressful Life Event

*Covariate effects.* The covariates were significantly associated with the distal outcome variables in the expected directions (Table 8, lower part). Females reported a higher symptom burden than males, with the caveat that this was a predominantly female sample (95%). Individuals with lower education had significantly more ED symptoms than those with higher education, whereas higher age implied significantly less symptoms of depression.

When examining PHQ depression and GAD anxiety as distal outcomes, the EDE-Q symptom score was included as a covariate in order to provide an adjustment in these analyses due to the case – control nature of the study sample. Having more eating disorder symptoms was positively associated with more depression and anxiety scores, as expected. Since EDE-Q also was correlated with gender, education, and age (higher scores for females, lower education, and younger age), these covariate effects canceled out and was overtaken by EDE-Q.

## Final adjusted distal outcome mean scores

The adjusted mean values of the distal outcome variables are given in Table 9.

*The SLE latent variable.* The final adjusted mean scores for all distal outcome variables (Table 9) showed an increase in the symptom burden when moving from class #1 (low exposure) through to class #4 (broad and high adversity). Post-hoc testing (not part of Table 9) showed a significant difference (*p* < 0.001) between class #1 and #2, and class #1 and #3 for ED diagnosis; between class #1 and #3, and class #1 and #4 for EDE-Q; between class #1 and #4 for PHQ depression, whereas no significant mean class differences were observed for GAD anxiety. Calculation of standardized mean difference (effect size, M = 0, SD = 1) showed highest SMD between class #1 and #4 with SMD’s equaling 0.49 (EDE-Q), 0.34 (PHQ-9) and 0.19 (GAD-7). These effect sizes were in the medium to low range.

*The latent protection variable (RSA/DERS).* As for the SLE class variable, moving to a higher RSA-DERS profile number, from #1 to #6, implied an increasing symptom burden. Moreover, the increase in symptom burden followed a stepped curve characteristic with minor differences between profile #1 and #2 (the two best profiles in terms of protection), an increased but roughly comparable symptom burden for profile #3 and #4, and finally, a further increased symptom burden for profile #5 and #6. Confidence intervals (99%) and post-hoc tests of these differences are described in the cells and notes of Table 9, respectively. In addition, some of the differences within steps (as categorized above) were also significantly different, e.g., profile #3 and #4, and #5 and #6 for PHQ depression. The SMD differences between profile #1 and #2 were minor across all distal outcomes (average = 0.13, range 0.10–0.16), but high between the combined profile #1 and #2 and the combined profile #3 and #4 scores (average = 0.65, range 0.54–0.80) and very strong between the combined profile #1 and #2 and the combined #5 and #6 scores (average = 1.55, range 1.40–1.71). The effect sizes related to the latent RSA-DERS factor were thus strong and substantially higher than for the latent SLE factor.

## Discussion

The current study examined how stressful life events (SLE) and protective resources (here measured as resilience and emotion regulation abilities), are expressed in a sample of individuals with and without a lifetime history of EDs. We used mixture modelling (latent class analysis) to identify how SLEs and protective resources, as well as their combination, are differently expressed in subgroups of the sample, as well as the associations of these classifications with lifetime ED diagnosis, ED symptoms, and associated symptoms of depression and anxiety. The LCA analyses revealed four classes based on participants’ exposure to SLEs. Although the majority of individuals belonged to classes with low to medium levels of exposure, around one quarter of the sample fell into classes characterized by high adversity or sexual assaults. The latent analysis of protective resources settled on 6 profiles differentiating individuals ranging from high to low levels of protection. Participants mainly differed in terms of quantitative levels of resources, except for some classes that had comparable levels of intrapersonal resources (e.g., personal and social competence) but various interpersonal levels of resources (i.e. family cohesion and social resources). The main finding from the final outcome model was that the latent variables for SLE exposure and protective resources significantly predicted levels of psychopathology and ED case status, with larger effects for protective resources. The relationship between SLEs and the psychopathology outcome data was not moderated by the protective resources classifications.

Investigating protective resources in relation to mental health outcomes provides an important addition to risk factor research. As there are likely both risk and protective factors influencing an individual’s vulnerability to develop psychopathology, the combinations of these factors are important to explore. To our knowledge, this is the first study to explore the nature of resilience factors using a mixture modelling approach in the context of EDs. A previous study investigating resilience among healthy adolescents with the same measure as in the current study mainly supported a four-profile solution that primarily differed in terms of quantity [60]. This is relatively comparable to the results of the present study as we also considered a four-profile solution because the addition of the two extra profiles offered minor improvements in model fit. However, they provided some extra differentiation with regard to family and social resources that we deemed substantial enough to warrant further exploration. Our analysis also included emotion regulation as part of the analysis. Similarly, recent studies conducting LPA analysis of emotion regulation profiles in individuals with or at risk for EDs have found three or four profiles clearly distinguishing emotion regulation and ED characteristics [14, 61]. Despite our study combining emotion regulation with resilience factors in the LPA, our prime finding was comparable to these previous studies by mainly supporting a quantitative differentiation with an increasing symptom burden for profiles characterized by lower protection. We also included individuals both with and without EDs, which might have influenced the class differentiation in our study. The extra differentiation we observed in terms of higher versus lower interpersonal and intrapersonal resilience factors is an interesting finding that calls for further scrutiny about how these factors are associated with ED pathology. In our study, individuals in the two profiles characterized by the lowest levels of protection had symptom levels that were within an ED clinical range regardless of SLE exposure, and within associated clinical range for depression and anxiety irrespective of ED pathology level. This indicates that individuals with low levels of protective resources, hence indicating a lower capacity for adaptation, more commonly have symptoms of mental disorders.

While the main focus of the current study was the associations between resilience factors and EDs, correlations were stronger between the RSA and measures of depression and anxiety symptoms than ED pathology. Consistent with this, the effect of the LPA variable for protective resources was also present in the models for depression and anxiety in addition to ED symptoms. The positive effects of having more protective resources available is thus not specific to ED symptomatology, but extends to a range of pathologies in line with previous studies showing that RSA is associated with both depression and anxiety across different contexts [36, 62]. While we cannot establish directional effects in a cross-sectional study, the results are consistent with previous empirical findings regarding resilience that suggest low protective resources as a vulnerability factor for developing maladaptive habits or cognitions that could translate into mental illness [35].

While the RSA defines resilience as not just an outcome, but a set of protective resources [63], other descriptions have defined it as an ability to «bounce back» after a trauma or stressful experience [64]. These different ways of conceptualizing resilience imply variations in how resilience is measured and interpreted. Despite having a good rationale for measuring resilience as protective resources, the understanding of which factors or resources to measure is far from clear-cut. Resilience is thus closely tied to the instruments that are used to measure it [65]. In relation to this, a systematic review by Windle, Bennett [66] identified no “gold standard” method for measuring resilience, but the RSA, as included in the current study, received high ratings in terms of adequately capturing the breadth of the construct, i.e., covering four intrapersonal domains (e.g., personal and social competence), as well as external inter-personal domains (i.e., family cohesion and social resources). The interpretations in the current study must thus be seen in light of the chosen measure and how it operationalizes the underlying concept.

In our study, we used a data-driven, person-centered approach to investigate the relationship between protective factors and SLEs in individuals with EDs. In previous conventional regression analytic studies, the RSA has been supported as a protective measure by moderating or dampening the negative effects of a stressor on an outcome [35, 67]. Similar findings have been reported in studies using other resilience measures assessing resilience as an outcome rather than a set of protective factors (i.e., the CD-RISC). For example, Yubero, de las Heras [68] reported that resilience moderated the relationship between chronic bullying and current well-being and Wingo, Wrenn [69] found that resilience moderated the association between trauma and depression. Thus, we had reason to expect comparable moderation effects, which we did not observe. The latent profiles of protective resources instead showed strong associations with the distal outcomes in this study; hence, the addition of a significant moderation effect would contribute less. Protective factors, such as personal competence and self-acceptance, have been associated with long-term quality of life in individuals with EDs [22], but are still relatively understudied within the ED literature. Since resilience has been highlighted as a possible important factor in ED recovery [70–72], this indicates a complex interplay between risk and protective factors that warrants further exploration in future longitudinal studies on the psychological functioning and outcome of ED patients.

Strengths of this study include the use of an exploratory person-centered approach. We incorporated latent variables of both potential risk and protective factors into the same model to explore relationships to EDs. We were able to extract separate classes/profiles of individuals, and all subsequent analyses using the latent variables were conducted within the LCA framework which does not overstate clustering accuracy by retaining measurement errors inherent in such classifications. This method allowed for individualized patterns of responses to be considered in the analysis. By including highly correlated SLE’s and protective factors in one model, which causes multi-collinearity problems in conventional regression analyses, we were able to shed light on individual differences in potential risk/protection profiles with regard to ED pathology and related symptom burden.

This study has some limitations. First, we did not measure protective resources prior to exposure to potential stressors, which means that resilience or emotion regulation resources may have been influenced by the participants’ current mental state and history of adversity. Longitudinal studies are needed to explicitly test the stability of these protective factors over time. However, given the high test–retest stability of the RSA in previous follow-up studies [15], the strong correlations with stable personality traits [34] and comparable findings in previous studies on stressful events or adversities [35, 67, 73], a similar protective role of these resources in the present study is likely. Second, our sample was predominantly female and we did not record information on race, ethnicity, or immigration status, precluding us from exploring these potential covariates further. Third, the sample sizes for some of the latent class within-group combinations were low and of possible low statistical power, which also prohibited stratified LCA analyses based on ED case or control status. However, having substantial heterogeneity in the sample data may also be considered an important premise [24], and contribute to identify latent classes that differentiate well between clinical and non-clinical cases. This was the case in the present study, showing a strong latent class differentiation for the ED case / non-case status variable (shown in Table 9). Fourth, all data was self-reported and relied on each individual’s memory of past events and accurate reporting of symptoms and descriptives. Finally, as the current study aimed to compare cases and controls with and without EDs, the sample is naturally biased towards specific subgroups and the sample must be viewed as a convenience sample not necessarily representative of the larger population.

## Conclusions

In this study, we investigated both potential risk and protective factors within a latent variable model for individuals with eating disorders. Notably, protective factors had a large effect on the pathology measures whereas the contribution of stressful life events were minor. Individuals with low availability of protective resources may be at a higher risk of maintaining maladaptation or psychiatric symptoms following illness or other stressful events causing such problems. Expanding this knowledge could be used to target preventative measures to facilitate resilience and lessen the burden of EDs and other mental health difficulties.

## Data Availability

The datasets used and/or analyzed during the current study are available from the corresponding author on reasonable request.

## Abbreviations

AN: Anorexia nervosa
BED: Binge-eating disorder
BN: Bulimia nervosa
DERS-SF: Difficulties in emotion regulation scale – Short form
ED: Eating disorder
EDE-Q: Eating disorder examination questionnaire
GAD: Generalized anxiety disorder scale
LCA: Latent class analysis
LPA: Latent profile analysis
PHQ: Patient health questionnaire
RSA: Resilience scale for adults
SLE: Stressful life event
